# Bimanual Force Variability and Chronic Stroke: Asymmetrical Hand Control

**DOI:** 10.1371/journal.pone.0101817

**Published:** 2014-07-07

**Authors:** Nyeonju Kang, James H. Cauraugh

**Affiliations:** Motor Behavior Laboratory, Applied Physiology and Kinesiology Department, University of Florida, Gainesville, Florida, United States of America; University of Ottawa, Canada

## Abstract

The purpose of this study was to investigate force variability generated by both the paretic and non-paretic hands during bimanual force control. Nine chronic stroke individuals and nine age-matched individuals with no stroke history performed a force control task with both hands simultaneously. The task involved extending the wrist and fingers at 5%, 25%, and 50% of maximum voluntary contraction. Bimanual and unimanual force variability during bimanual force control was determined by calculating the coefficient of variation. Analyses revealed two main findings: (a) greater bimanual force variability in the stroke group than the control group and (b) increased force variability by the paretic hands during bimanual force control in comparison to the non-paretic hands at the 5% and 25% force production conditions. A primary conclusion is that post stroke bimanual force variability is asymmetrical between hands.

## Introduction

Variability abounds when chronic stroke individuals attempt to control force production. Post stroke deficits in bimanual force control are common, especially in activities of daily living (e.g., buttoning a blouse/shirt or buttering a piece of bread). Movement variability arises from both bimanual and unimanual motor actions. However, bimanual variability does not equal the summation of variability from paretic and non-paretic hands.

Previous bimanual force control studies demonstrated impaired capabilities post stroke [1]–[3]. Bimanual force variability, as indicated by the coefficient of variation (CV), produced by individuals in the chronic stage of recovery post stroke was greater than an age-matched control group [1], [2]. Lodha and colleagues investigated bimanual variability by manipulating three levels of force output (e.g., 5%, 25%, and 50% of maximum voluntary contraction; MVC) [2], [4]. Dissociating the force produced by each hand revealed an asymmetry between hands. The paretic hands produced less force than the non-paretic hands across the three force levels [2], [4], [5]. Moreover, the bimanual coordination findings indicated that the non-paretic hands regulated the combined bimanual force production via an increased time lag between hands [2]. Together, these findings suggested that the greater bimanual force variability post stroke may be attributed to decreased force output in the paretic hands as well as impaired function of the non-paretic hands.

Unfortunately, the above explanations do not answer a critical stroke motor recovery question about bimanual movements: Does force control asymmetry between hands show up in force variability data? Even though previous unimanual force control studies reported higher force variability in the paretic hands than the non-paretic hands [3], [6]–[8], no study has determined whether force variability between the two hands is different during bimanual force control. Moreover, brain imaging findings revealed that less cortical activity in the ipsilesional hemisphere increased force variability during unimanual paretic hand control [9]. Further, the deficits in bimanual force control post stroke are associated with unbalanced inter-hemispheric inhibition between hemispheres (e.g., greater inter-hemispheric inhibition in the contralesional hemisphere than the ipsilesional hemisphere) via callosal connections contributing to decreased cortical activity in the ipsilesional hemisphere [10], [11]. Consequently, the unbalanced inhibitory and excitatory activities between hemispheres post stroke may induce asymmetrical force variability between hands during bimanual force control. Indeed, force variability asymmetry may interfere with bimanual force control.

This knowledge is important because rehabilitation programs could structure their protocols with a clear understanding of bimanual force control as well as force variability. Earlier bimanual training focused on recovery of bimanual resultant function [12], [13]. However, if force variability asymmetry causes impairments in bimanual coordination post stroke, then rehabilitation goals should focus on minimizing the force variability asymmetry between hands. Thus, the primary purpose of this study was to investigate force variability produced by the paretic and non-paretic hands during bimanual force control to determine hand influences on bimanual force variability in chronic stroke. We hypothesized that the force variability produced by paretic hands would be greater than non-paretic hands during bimanual force control across force levels. Further, the greater force variability by the paretic hands than non-paretic hands would be associated with an increase in bimanual force variability.

## Methods

### Ethics Statement

The University of Florida's Institutional Review Board approved the protocol and informed consent form involved in this study. Prior to beginning testing, all participants read and signed an informed consent.

### Participants

Volunteers included nine stroke individuals [mean age = 64.2 years (*SD* = 18.8); 6 females and 3 males] and nine age-matched controls [mean age = 67.6 years (*SD* = 13.8); 3 females and 6 males]. Additional characteristics of the stroke group follow: (a) impaired hemisphere: 6 left and 3 right; (b) stroke type: 8 ischemic and 1 hemorrhagic; (c) mean time since stroke = 4.6 years (*SD* = 4.4); and (d) mean hand function score on the Stroke Impact Scale (version 3.0) = 72.2 (*SD* = 21.4) [14]. Three criteria for testing the stroke participants included: (a) unilateral stroke at least 6 months before starting testing; (b) voluntary movement from 80° of flexion to 10° of wrist extension; and (c) intact cognitive function (Mini Mental State Examination score >23) [15]. Exclusion criteria involved additional neurological or musculoskeletal disorders.

### Bimanual Force Control Task: Wrist and Fingers Extension

**Figure 1:**
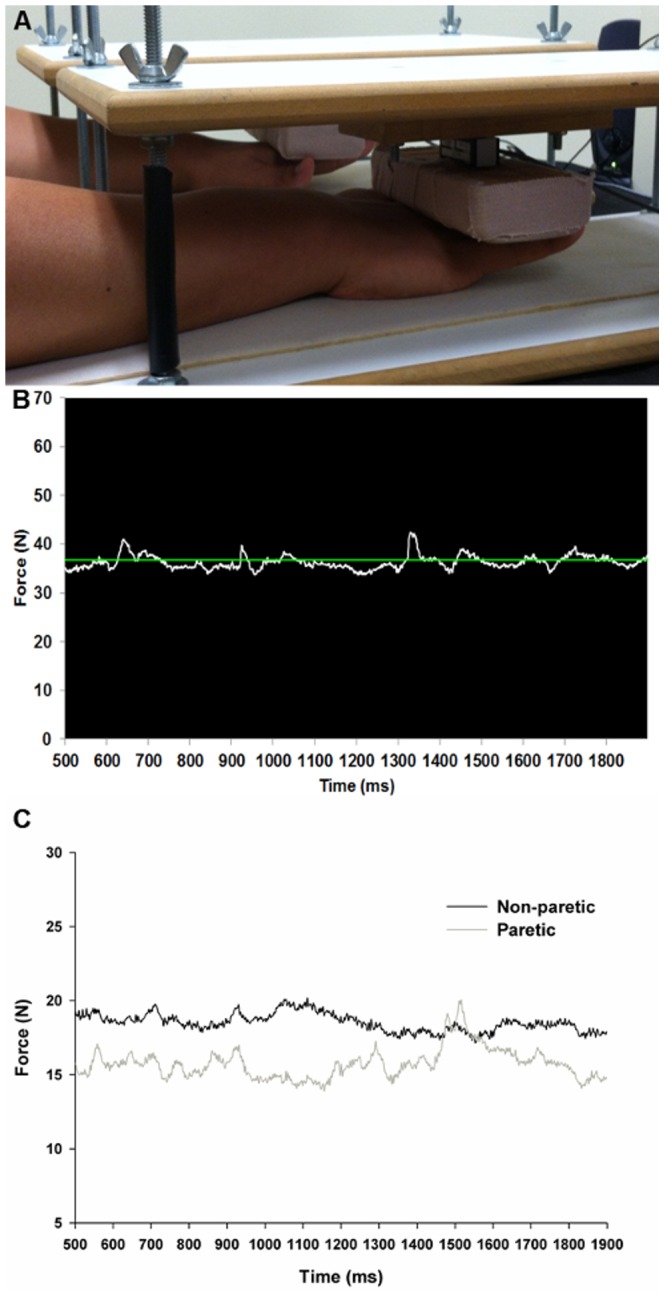
Bimanual force control task. (A) Wrist and fingers extension position. (B) Example of visual display during the task. (C) Representative force signals in the paretic and non-paretic hands at 25% of MVC.

Based on previous findings, a wrist and fingers extension task was used for the bimanual isometric force control at 5%, 25%, and 50% of MVC [1], [16]. Participants were seated 78 cm away from 43.2 cm monitor (1024×768 pixels, 100 Hz refresh rate) and placed their left and right forearms on the table while maintaining shoulder flexion (15–20°) and elbow flexion (20–40°) in comfortable positions. Each extended hand was placed under separate padded platforms (embedded with force transducers; MLP-75, Transducer Techniques, 4.16×1.27×1.90 cm, range = 75 lbs, 0.1% sensitivity) and the height of each platform was adjusted based on each participant's hand thickness (Figure 1A).

The bimanual force control task required participants to extend their wrist and fingers upward against the padded platforms. Before testing began, everyone performed three bimanual MVC trials. The mean values determined three submaximal target levels: 5%, 25%, and 50% of MVC. The force production level for each participant indicated the maximum level of combined bimanual force. During the 20 s bimanual force control task, participants tried to match their combined bimanual forces (summed forces produced by both hands) to each of the three randomly presented target levels (see Figure 1B).

Considering that more visual information decreased force variability, we normalized the amount of visual information across our three target force levels with a constant visual angle [17]–[19]. As seen in Equation 1, we manipulated the height of force fluctuation character for each force level [19], [20]. The visual angle was set at a constant 1° across the force levels (Equation 2). Regulating the visual gain (pixels/N) maintained the constant visual angle (5% of MVC: 13 pixels/N and 25% and 50% of MVC: 8 pixels/N). Further, calculating the height of force fluctuation character involved six times each force standard deviation for each target level (5% of MVC: SD = 0.3 N and 25% and 50% of MVC: SD = 0.5 N). This standardized method is consistent with previous studies [19], [20].
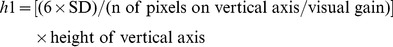
(1)

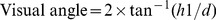
(2)
*h*1 indicates height of force fluctuation character and *d* (i.e., 0.78 m) indicates a distance from monitor to eye level.

A 15LT Grass Technologies Physio-data Amplifier System (Astro-Med Inc.) with an excitation voltage of 10 V and a gain of 200 amplified the force signals. A 16-bit analog-to-digital converter (A/D; NI cDAQ-9172+NI 9215) collected the force signals at 100 Hz of sampling rate (minimum detected force unit = 0.0016 N). A custom LabVIEW program (National Instruments, Austin, USA) managed the bimanual force control tasks. Offline analyses included a custom Matlab program (Math Works Inc., Natick, USA).

### Data Analyses

Initial force data analysis involved removing the first 5 s and last 1 s from the original signals. Next, we eliminated noise in the force signals by submitting the middle 14 s of data for each trial to a bidirectional fourth-order Butterworth filter (cut off frequency = 20 Hz). Finally, the force data were detrended to exclude any drift away from target levels [17].

Absolute measures (e.g., standard deviation and root mean square error) for force variability are not normalized by force production levels, and consequently, can be affected by an individual's ability to generate force production. To minimize the effect of the magnitude of forces on force variability, we calculated CV normalized by the level of force production. The CV values of the combined bimanual force (i.e., SD/mean force produced by two hands×100) represented bimanual force variability. To quantify the CV produced by each hand during bimanual force control, we calculated the mean force produced by each hand: (a) paretic and non-paretic hands for the stroke group and (b) non-dominant and dominant hands for the control group. Representative force signals for the paretic and non-paretic hands are shown in Figure 1C. A two-way mixed design ANOVA (Group×Force Level: 2×3) analyzed the mean data for the CV of combined bimanual forces. For the CVs produced by each hand, we submitted the mean values to a three-way mixed design ANOVA (Group×Hand×Force Level: 2×2×3). Post hoc analyses followed Tukey-Kramer's procedure.

Despite normalizing the CV by the level of mean force production, Sosnoff and Newell [21] reported that greater force variability can be affected by an impaired ability to generate force production. Thus, we determined mean force production between groups and two hands during the task. The mean force production by each hand was submitted to a three-way mixed design ANOVA (Group×Hand×Force Level: 2×2×3).

A linear regression analysis was conducted to compare the force variability produced by each hand during bimanual force control to the bimanual force variability at the 5%, 25%, and 50% of MVC for the two groups. This analysis involved identifying an explanatory variable: the difference in CV for each group separately (i.e., CV by the paretic hands minus CV by non-paretic hands for the stroke group; CV by the non-dominant hands minus CV by dominant hands for the control group). Bimanual force variability was the dependent variable.

A positive relationship (β>0) between the explanatory and dependent variables indicated greater force variability by the paretic hands (or non-dominant hands for the control group) than the non-paretic hands (or dominant hands for the control group) was associated with increased bimanual force variability and vice versa. In contrast, a negative relationship (β<0) revealed that less force variability by the paretic hands (or non-dominant hands for the control group) than the non-paretic hands (or dominant hands for the control group) was associated with increased bimanual force variability and vice versa. *R* ^2^ measured the goodness-of-fit of the model [22]. For all statistical tests, alpha level was set at 0.05.

## Results

### Mean Force Production

To determine whether the levels of force production between groups and hands differed, we performed a Group×Hand×Force Level (2×2×3) mixed design ANOVA on mean force production by each hand. The analysis did not reveal any differences in mean force production between groups or hands. The mean forces produced by each hand collapsed across force levels were: (a) paretic hands: *M* = 22.0 N (*SE* = 3.5); non-paretic hands: *M* = 26.5 N (*SE* = 3.9) and (b) non-dominant hands: *M* = 29.6 N (*SE* = 4.3); dominant hands: *M* = 27.0 N (*SE* = 4.1). These findings confirm that the level of mean force did not differ between groups or hands.

### Force Variability: Coefficient of Variation

A two-way mixed design ANOVA on the bimanual force variability (CV) revealed two significant main effects: (a) group [*F*(1, 16) = 8.76; *p* = 0.009; η^2^ = 0.35] and (b) force level [*F*(2, 32) = 8.07; *p* = 0.001; η^2^ = 0.34]. The stroke group (*M* = 4.0%; *SE* = 0.5%) showed significantly greater bimanual force variability than the control group (*M* = 2.4%; *SE* = 0.2%). Concerning the force level main effect, the bimanual force variability for the 5% condition (*M* = 4.2%; *SE* = 0.5%) was significantly greater than at the 25% condition (*M* = 2.1%; *SE* = 0.2%).

**Figure 2:**
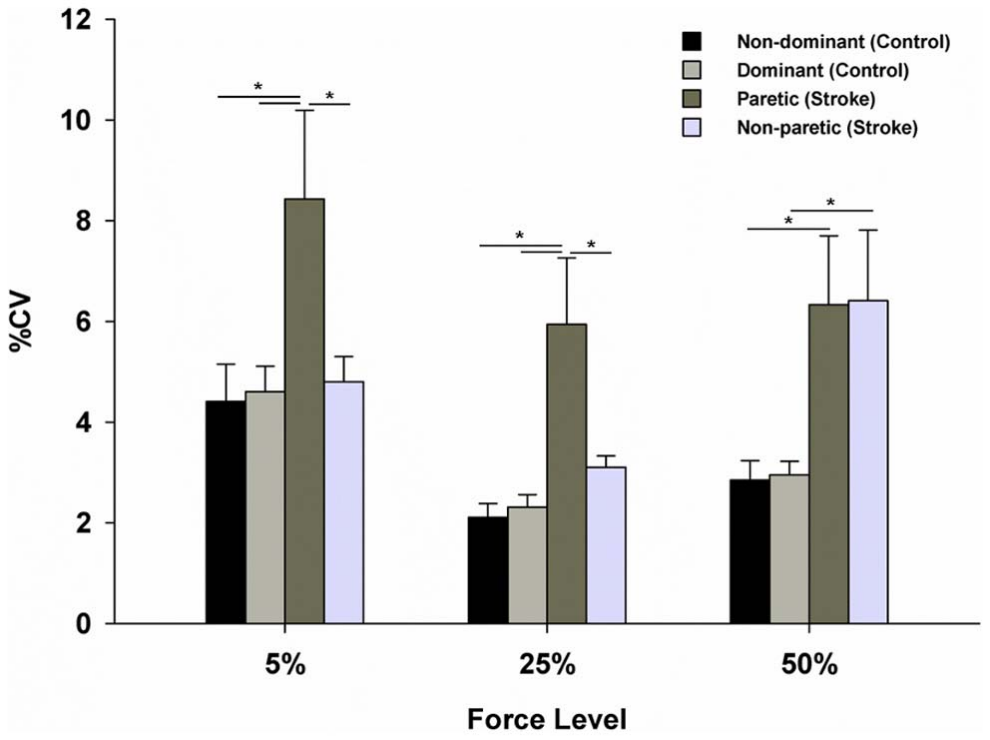
Mean coefficient of variation produced by each hand during bimanual force control. (*M*±*SE*). *Asterisk* (*) indicates significant difference (*p*<0.05).

Moreover, to determine force variability (CV) between two hands during bimanual force control, a three-way mixed design ANOVA on the force variability by each hand was performed. The analysis revealed a significant Group×Hand×Force Level (2×2×3) interaction [*F*(2, 32) = 6.71; *p* = 0.004; η^2^ = 0.30; Figure 2]. Post hoc analyses indicated that force variability by the paretic hands in the stroke group was significantly greater than the non-paretic hands as well as the non-dominant and dominant hands in the control group at 5% and 25% of MVC. At 50% of MVC, the paretic and non-paretic hands showed significantly greater force variability than the non-dominant and dominant hands in the control group. In contrast, the control group showed similar force variability between the non-dominant and dominant hands across the three force levels.

### Bimanual Force Variability versus Asymmetrical Force Variability

**Figure 3:**
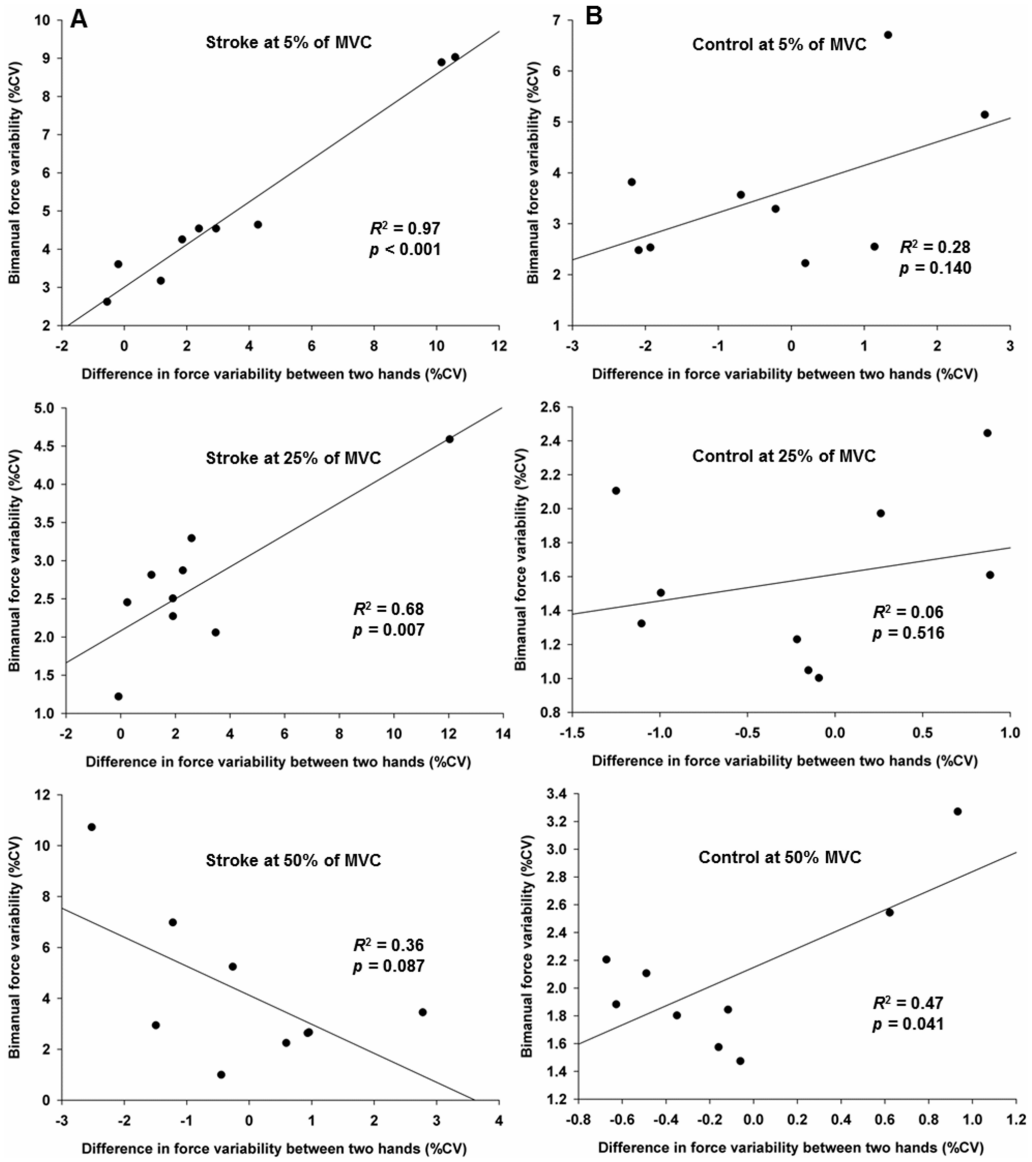
Regression models showing relationship between difference in force variability and bimanual force variability. Difference in force variability for each group (paretic minus non-paretic hands for stroke group and non-dominant minus dominant hands for control group) (A) For the stroke group, greater force variability by the paretic hands than the non-paretic hands was associated with increased bimanual force variability at 5% of MVC and 25% of MVC. (B) For the control group, less force variability by the non-dominant hands than the dominant hands was associated with decreased bimanual force variability at 50% of MVC.

Determining the force variability contributions of each hand to bimanual force variability involved conducting a linear regression analysis on the three force levels for each group. Analysis of the stroke group's data revealed greater force variability (CV) in the paretic hands than the non-paretic hands. The greater force variability was associated with an increased bimanual force variability at the two lower force levels. For the 5% condition, the bimanual CV equation 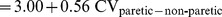 (*p*<0.001, *r* = 0.98, *R* ^2^ = 0.97; see Figure 3A). For the 25% condition, the bimanual CV equation 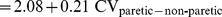 (*p* = 0.007, *r* = 0.82, *R* ^2^ = 0.68; see Figure 3A). Moreover, the force variability by the paretic hands was greater than the non-paretic hands in 78% of the stroke participants at the 5% condition and in 89% at the 25% condition.

In contrast, most individuals in the control group produced similar force variability between hands at the two submaximal force levels. At the 50% condition, the control participants showed decreased bimanual force variability. The non-dominant hands force variability values were less than the dominant hands: the bimanual CV equation 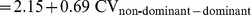 (*p* = 0.041, *r* = 0.69, *R* ^2^ = 0.47; see Figure 3B). Moreover, seven out of nine control participants at the 50% condition, displayed force variability by non-dominant hands was less than the dominant hands.

## Discussion

The purpose of this study was to investigate force variability generated by both the paretic and non-paretic hands during bimanual force control. Analyses revealed two primary findings: (a) greater variability of the combined bimanual forces in the stroke group versus the control group and (b) higher CV in the paretic hands than the non-paretic hands and the control group (non-dominant and dominants hands) at 5% and 25% of MVC.

The greater bimanual force variability found in the stroke group than the control group is consistent with previous findings [1], [2]. Moreover, new evidence from the current study revealed more impaired force control variability in the paretic hands than the non-paretic hands during bimanual force control (i.e., force variability asymmetry). A previous unimanual force control study revealed greater force variability in the paretic hands than the non-paretic hands [6]. Thus, force variability asymmetry between hands post stroke may occur regardless of movement conditions (e.g., unimanual and bimanual movements). Indeed, at 5% and 25% of MVC force variability by the paretic hands was greater than the non-dominant and dominant hands in the control group. These findings indicate that the asymmetrical force variability between hands during bimanual force control is a predominant characteristic of motor impairments post stroke.

Consistent with our hypothesis, regression analyses for the stroke group revealed that the greater force variability by the paretic hands than the non-paretic hands was strongly associated with an increase in bimanual force variability at the 5% and 25% of MVC. These findings support the supposition that bimanual force control is more affected by the paretic hands control than non-paretic hands at the submaximal target levels. Consequently, quantifying bimanual force control variability for the two lowest MVC conditions (5% and 25% of MVC) provides motor impairment evidence in the paretic hands as well as bimanual motor function [23].

Earlier bimanual stroke studies demonstrated less force production by the paretic hands than non-paretic hands and emphasized that restoring similar force production between the hands is an important goal in stroke rehabilitation [2], [4]. In the present study, despite the relatively comparable forces produced between the hands, the force variability asymmetry during the bimanual force control task persisted in stroke survivors. A possible mechanism underlying force variability asymmetry with comparable force outputs between hands involves impairments in controlling neuromotor drive and noise. Massie and colleagues reported that force variability (CV) in paretic hands decreased after passive repetitive transcranial magnetic stimulation (i.e., receiving stimulation during relaxation) with a reduction in muscle activities [24]. However, the level of force outputs produced by the paretic hands did not change across test sessions. These findings indicate that an ability to modulate neuromotor drive and noise from the motor area may differentiate force variability modulation while participants are executing a constant force [24], [25]. Moreover, earlier studies demonstrated that increased force levels did not directly reduce variability in motor outputs [26], [27]. Thus, a reasonable suggestion is that when determining force production capabilities one should quantify force variability by the paretic hands during bimanual force control.

Motor control performed by healthy individuals shows that one hand is not always equivalent to the other hand when executing bimanual movements. Brain imaging studies revealed that activation of motor areas in the dominant hemisphere was greater than in the non-dominant hemisphere [28], [29]. The unequal amount of brain activity between hemispheres may cause asymmetrical motor control between hands [22], [29], [30]. These studies reported that a certain level of motor control asymmetry between two hands may exist during bimanual movements and positively influence bimanual coordination. However, as seen in stroke survivors, an excessive motor control asymmetry between two hands interfered with bimanual motor control. Perhaps, more unbalanced brain activity between hemispheres post stroke results in an asymmetrical force control between hands during bimanual force control [11]. Consequently, the present findings indicate that minimizing force variability asymmetry between paretic and non-paretic hands during bimanual force control provides additional meaningful clinical information about progress toward motor recovery.

For the 50% level of MVC task, force variability by the non-paretic hands increased as much as the paretic hands for the stroke group and the force variability by the paretic and non-paretic hands was greater than the non-dominant and dominant hands for the control group. Accumulated findings indicate that the higher target level may have increased compensation by the non-paretic hands in stroke survivors. However, given that stroke survivors typically have motor deficits on their ipsilesional side as well [31]–[33], force variability by the non-paretic hands in the stroke group was greater than the dominant hands in the control group. On the contrary, the control group successfully decreased bimanual force variability at 50% of MVC by increasing the role of dominant hands (i.e., less variability by the non-dominant hands than the dominant hands associated with reduced bimanual force variability). Thus, bimanual force variability at the higher force level appears to reflect impaired ipsilesional hand function post stroke.

In conclusion, the current stroke study clearly revealed asymmetrical force variability between the two hands at 5% and 25% of MVC. Moreover, the greater force variability by the paretic hands than the non-paretic hands increased bimanual force variability. These novel findings suggested that the impaired force control capability in the paretic hands during bimanual force control is a prominent characteristic of motor deficits post stroke. Moreover, the amount of bimanual force variability in stroke survivors reflects their paretic hand control capabilities at the lowest and medium target force levels. An extension of present study will investigate force variability produced by the paretic and non-paretic hands during unimanual and bimanual movement conditions to determine whether the asymmetrical force variability between two hands during bimanual movements is greater than during unimanual movements.
